# Relationship between lipoproteins, thrombosis, and atrial fibrillation

**DOI:** 10.1093/cvr/cvab017

**Published:** 2021-01-23

**Authors:** Wern Yew Ding, Majd B Protty, Ian G Davies, Gregory Y H Lip

**Affiliations:** 1 Liverpool Centre for Cardiovascular Science, University of Liverpool and Liverpool Heart & Chest Hospital, William Henry Duncan Building, 6 West Derby Street, Liverpool L7 8TX, UK; 2 Systems Immunity University Research Institute, Cardiff University, Cardiff, UK; 3 Research Institute of Sport and Exercise Science, Liverpool John Moores University, Liverpool, UK; 4 Department of Clinical Medicine, Aalborg Thrombosis Research Unit, Aalborg University, Aalborg, Denmark

**Keywords:** Atrial fibrillation, Lipids, Lipoproteins, Low-density lipoprotein, Very low-density lipoprotein, High-density lipoprotein, Oxidized lipoprotein, Lipoprotein(a), Incidence, Haemostasis, Thrombosis, Thromboembolism, Stroke

## Abstract

The prothrombotic state in atrial fibrillation (AF) occurs as a result of multifaceted interactions, known as Virchow’s triad of hypercoagulability, structural abnormalities, and blood stasis. More recently, there is emerging evidence that lipoproteins are implicated in this process, beyond their traditional role in atherosclerosis. In this review, we provide an overview of the various lipoproteins and explore the association between lipoproteins and AF, the effects of lipoproteins on haemostasis, and the potential contribution of lipoproteins to thrombogenesis in AF. There are several types of lipoproteins based on size, lipid composition, and apolipoprotein category, namely: chylomicrons, very low-density lipoprotein, low-density lipoprotein (LDL), intermediate-density lipoprotein, and high-density lipoprotein. Each of these lipoproteins may contain numerous lipid species and proteins with a variety of different functions. Furthermore, the lipoprotein particles may be oxidized causing an alteration in their structure and content. Of note, there is a paradoxical inverse relationship between total cholesterol and LDL cholesterol (LDL-C) levels, and incident AF. The mechanism by which this occurs may be related to the stabilizing effect of cholesterol on myocardial membranes, along with its role in inflammation. Overall, specific lipoproteins may interact with haemostatic pathways to promote excess platelet activation and thrombin generation, as well as inhibiting fibrinolysis. In this regard, LDL-C has been shown to be an independent risk factor for thromboembolic events in AF. The complex relationship between lipoproteins, thrombosis and AF warrants further research with an aim to improve our knowledge base and contribute to our overall understanding of lipoprotein-mediated thrombosis.

## 1. Introduction

Atrial fibrillation (AF) is a multi-systemic condition that is associated with serious complications including thromboembolism, dementia, and heart failure, resulting in impaired quality of life, significant morbidity, and increased mortality.^1–5^ The prevalence of AF rises with age and concomitant comorbidities.^6^^,^^7^ At present, there is an upward trajectory to the global incidence and prevalence of AF.^8^^,^^9^ Indeed, every individual has a one-in-four lifetime risk of developing this condition,^10^^,^^11^ with a greater burden amongst those with risk factors.^12^ By 2060, it is projected that at least 17.9 million people in Europe will be affected by AF.^13^^,^^14^

The mechanism by which AF occurs is complex but has previously been described in detail.^15^ Management of patients with the condition is primarily focused on the prevention of thromboembolism due to the presence of a prothrombotic state with this arrhythmia. The prothrombotic or hypercoagulable state in AF occurs as a result of multifaceted interactions, known as Virchow’s triad of hypercoagulability, structural abnormalities, and blood stasis.^16^ Despite considerable research in this area, the precise mechanisms by which AF contributes to a prothrombotic state remains ill-defined.

There is emerging evidence that lipoproteins are implicated in thrombogenesis, beyond their traditional role in atherosclerosis. In this review, we provide an overview of the various lipoproteins and explore their relationship with AF, haemostasis, and the potential contribution to thrombogenesis.

## 2. Lipoproteins

**Figure 1:**
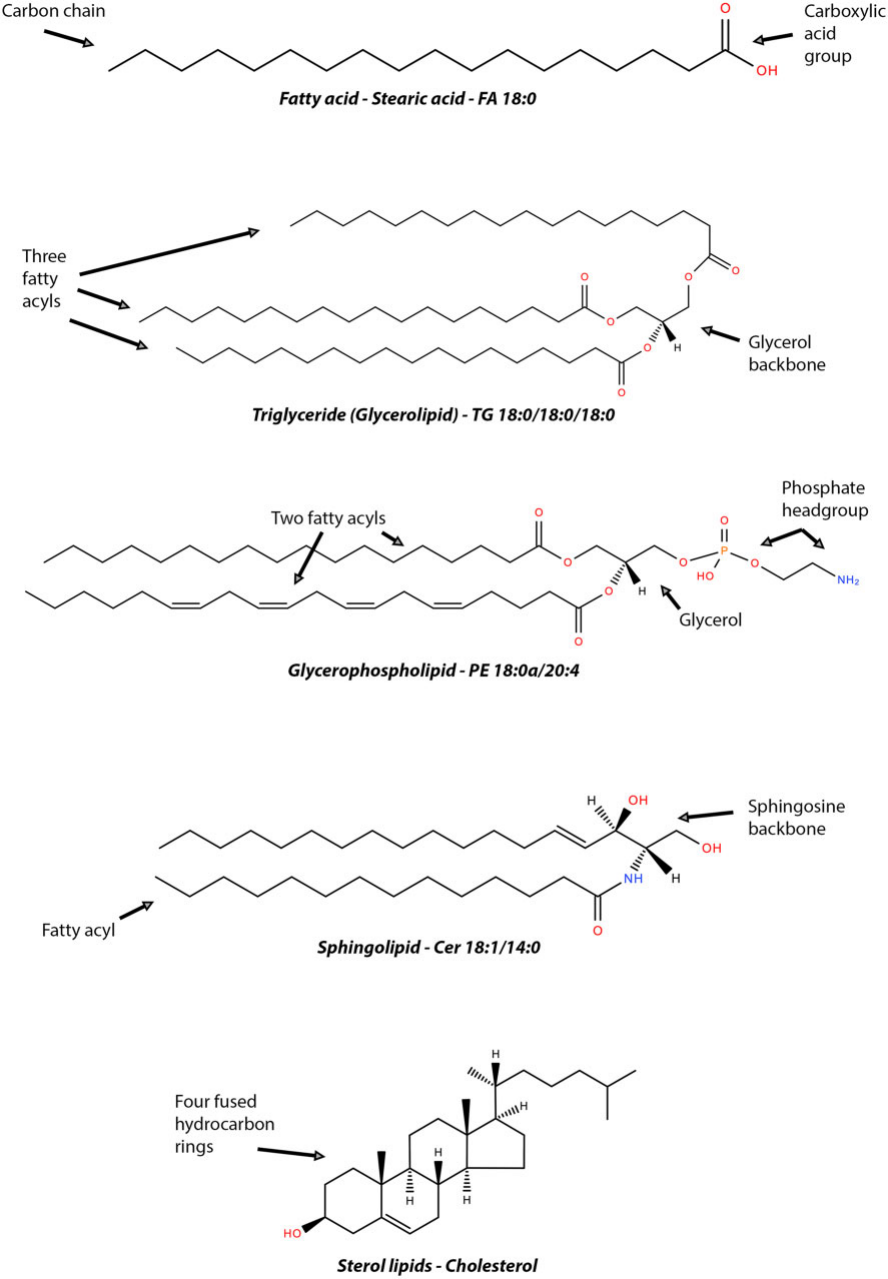
Representative schematic of lipid subtypes. Example structures from each LIPID MAPS category of lipids are shown in this figure highlighting their structural features. Fatty acids (FA), which may be saturated or unsaturated, form the basic building blocks of lipids, with each class having specific defining feature. Chemical structures are from PubChem and LIPID MAPS.

Lipids (also known as ‘fat’) are naturally occurring compounds serving numerous biological functions including the formation of plasma membranes or signalling molecules, and as a source of energy. They exist in several forms including free fatty acids, glycerolipids (GL), glycerophospholipids (GPL), sphingolipids, and sterol lipids. Each of these lipid subtypes have different molecular structures and basic properties (*Figure 1*). As a brief overview, fatty acids form the fundamental category of biological lipids and therefore the basic building blocks of more complex lipids. Their chemistry consists of a hydrocarbon chain with a terminal carboxylic acid group and may be defined as saturated or unsaturated depending on the maximum possible number of bonds or hydrogen atoms.^17^^,^^18^ GL consist of a single glycerol molecule which acts as the backbone for attachment to fatty acid chains. The most relevant example of GL are triglycerides (TG), which contain three fatty acid chains and play an important role in metabolism as energy sources and sources of dietary fat.^18^^,^^19^ Sterol lipids consist of four fused rings of hydrocarbon to which other molecules attach. A major type of sterol lipid is cholesterol which serves as a precursor for the synthesis of other steroids as well as serving as structural support for plasma membranes.^20^^,^^21^ Dietary cholesterol is often stored and transported in the form of a cholesterol ester (CE), which chemically represents a cholesterol molecule joined to a fatty acid via an ester bond.^22^

**Figure 2:**
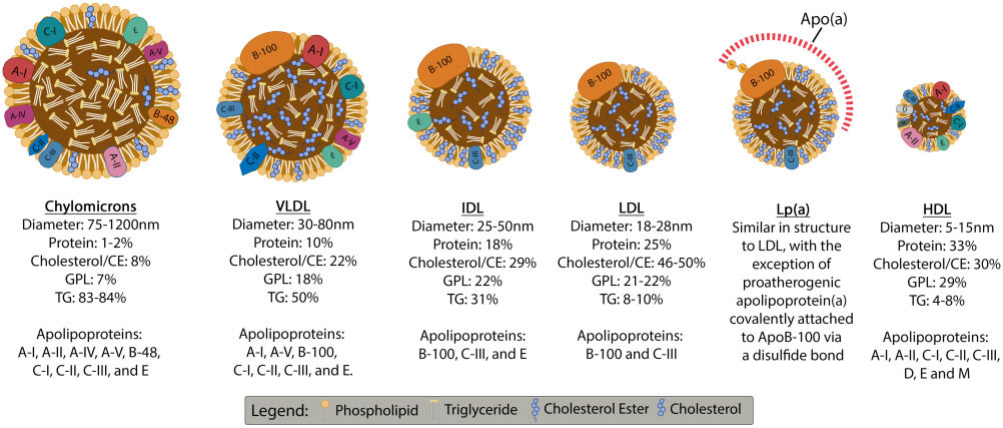
Lipoprotein types and structures. Representative description of typical diameter, content and apolipoprotein constituents of different lipoprotein classes.^23^ Created using Biorender.com. ApoB-100, apolipoprotein B100; CE, cholesterol ester; GPL, glycerophospholipids; HDL, high-density lipoprotein; IDL, intermediate-density lipoprotein; LDL, low-density lipoprotein; Lp(a), lipoprotein(a); TG, triglycerides; VLDL, very low-density lipoprotein.

One common feature that lipids share as a group is their insolubility in water. Consequently, they must be transported with proteins in the circulation (‘lipoproteins’).^23^ Lipoproteins are complex structures consisting of a central hydrophobic core primarily composed of CE and TG which is surrounded by a hydrophilic membrane comprising of GPL, free cholesterol, and apolipoproteins.^23^^,^^24^ There are several types of lipoproteins based on size, lipid composition, and apolipoprotein category, namely: chylomicrons, very low-density lipoprotein (VLDL), low-density lipoprotein (LDL), intermediate-density lipoprotein, and high-density lipoprotein (HDL). When elevated, all lipoproteins confer a pro-atherogenic risk, apart from HDL which is anti-atherogenic.^23^ Each lipoprotein contains numerous types of lipid species and proteins, whose composition varies even between individual lipoproteins of the same type (*Figure 2*).

**Figure 3:**
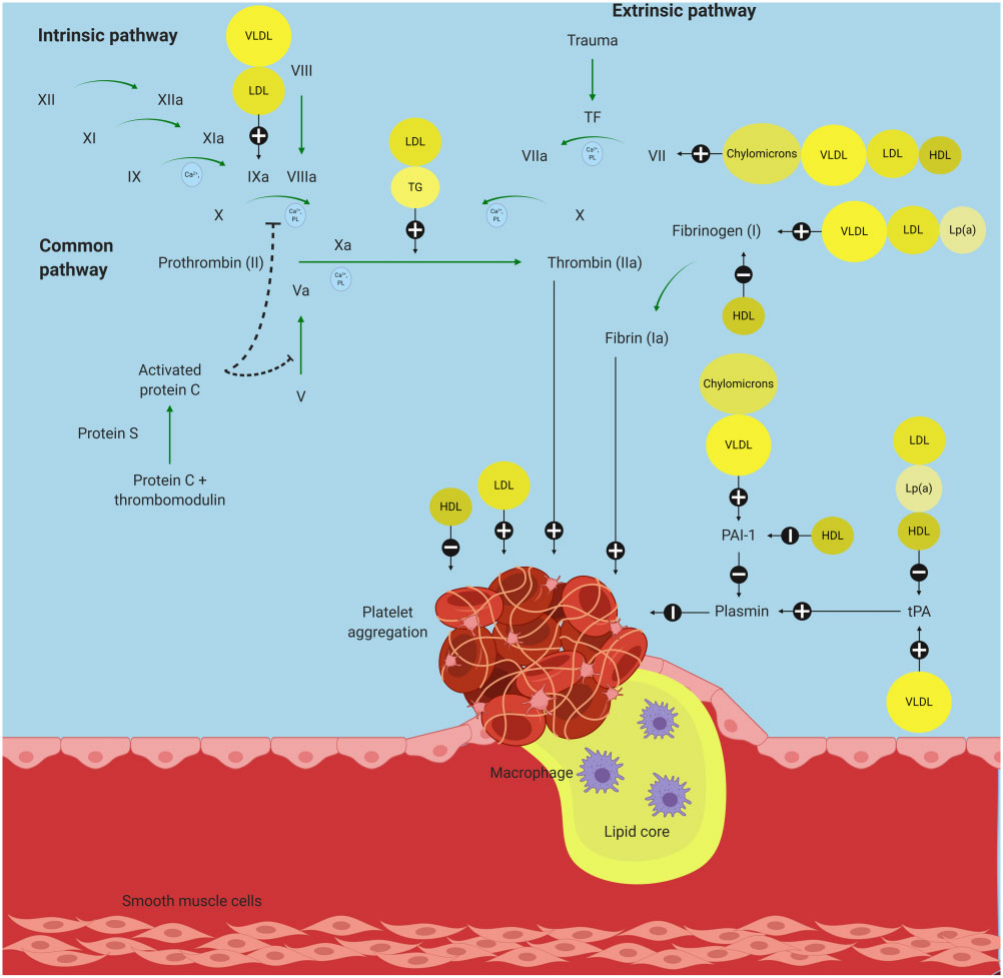
Effects of lipoproteins on haemostasis. Created using Biorender.com. HDL, high-density lipoprotein; LDL, low-density lipoprotein; Lp(a), lipoprotein(a); PAI-1, plasminogen activator inhibitor-1; TF, tissue factor; TG, triglycerides; tPA, tissue plasminogen activator; VLDL, very low-density lipoprotein.

LDL is the main transporter for cholesterol in the circulation and every LDL particle contains one apolipoprotein B100 molecule. LDL exists in a spectrum that varies in size and density with the three major density subclasses being small dense LDL (sdLDL), intermediate LDL, and large buoyant LDL.^25^ Small dense LDLs are considered more atherogenic and pro-coagulant compared to the other subtypes of LDL for various features as they have decreased affinity for LDL receptors and hence remain longer in the circulation, more readily enter the arterial intima where they are engulfed by macrophages to become foam cells, and are more susceptible to oxidation than its larger counterpart.^26–28^ There is also increasing evidence that the number of ApoB-rich particles or the concentration of apolipoprotein B may contribute to atherogenic risk.^29^

Modern lipidomic techniques, with the aid of liquid chromatography coupled to mass spectrometry, have allowed for detailed characterization of the LDL lipidome.^30^ This has revealed over 300 different lipid species residing within the interior or phospholipid membrane of the LDL particle. Each of these may have specific associations with various pathologies and interactions with traditional risk factors, thereby adding to its complexities.^31^^,^^32^ Oxidative modification of LDL, predominantly by non-enzymatic processes, leads to the formation of oxidized LDL (OxLDL) particles. These particles have altered structure and content, containing oxidized proteins and lipids (particularly GPL), and leading to a more atherogenic phenotype.^33^ Furthermore, the susceptibility of LDL to aggregation and proteoglycan binding has provided a deeper insight into the atherogenicity of LDL.^34^

Lipoprotein(a) [Lp(a)] is a specialized form of LDL assembled in the liver from LDL and apolipoprotein(a) attached to apolipoprotein B100 via a disulphide bridge (*Figure 2*).^35^ Lp(a) has been implicated in atherogenesis by enhancing endothelial cell adhesion and molecule expression, promoting the formation of foam cells by binding to macrophages with high affinity and interfering with vascular permeability.^36^ Furthermore, the Lp(a) constituent, apolipoprotein(a), shares many structural similarities with plasminogen which has been reported to cause interference with the physiological fibrinolysis process and to contribute to a prothrombotic phenotype.^37^

## 3. Lipoproteins and atrial fibrillation

### 3.1 The paradoxical inverse relationship between cholesterol and the incidence of AF

The association between serum cholesterol and coronary heart disease has been described since early 1964.^38^ There is an increased risk of coronary heart disease with elevated total cholesterol (TC) and LDL cholesterol (LDL-C), and reduced HDL cholesterol (HDL-C) levels.^39^^,^^40^ A longitudinal analysis over a 35-year period of patients from the Framingham study confirmed that long-term exposure to these lipid abnormalities led to a greater risk of atherosclerotic cardiovascular disease and mortality.^41^ Moreover, both the LDL particle and LDL-C are now considered causal for atherosclerotic cardiovascular disease.^42^ In turn, atherosclerotic disease is an established independent risk factor for incident AF.^43^^,^^44^ As such, elevated levels of TC and LDL-C may have been expected to increase the risk of incident AF. However, current evidence does not support this and in contrast, several well-conducted observational studies have described a paradoxical inverse relationship between TC and LDL-C, and incident AF (*Table 1*).

**Table 1:** Impact of lipoprotein abnormalities on incidence or prevalence of atrial fibrillation

Author, year (ref)	Study type	Population	*n*	Follow-up (months)	Finding(s) in relation to incidence or prevalence of AF
Harrison, 2020^45^	Prospective	Community-based cohort	13 724	NA	↑ TC: PR 0.61 (95% CI 0.49–0.75) ↑ LDL-C: PR 0.60 (95% CI 0.48–0.75) ↑ HDL-C: PR 0.58 (95% CI 0.46–0.74) ↑ non-HDL-C: PR 0.63 (95% CI 0.51–0.78) ↑ LDL-C/HDL-C ratio: PR 0.75 (95% CI 0.61–0.94)
Xue, 2019^46^	Prospective	STEMI	985	31	↑ TC: HR 0.54 (95% CI 0.32–0.90) ↑ LDL-C: HR 0.56 (95% CI 0.31–1.00) TG or HDL-C not found to be risk factors
Choe, 2018^47^	Retrospective	Population-based cohort	22 886 661	65	↑ TG: HR 1.12 (95% CI 1.12–1.13) ↑ HDL: HR 1.24 (95% CI 1.23–1.25)
Li, 2018^48^	Prospective	Community-based cohort	88 785	85	↑ TC: HR 0.60 (95% CI 0.43–0.84) ↑ LDL-C: HR 0.60 (95% CI 0.43–0.83) TG or HDL-C not found to be risk factors
Mourtzinis, 2018^49^	Retrospective	Hypertensive	51 020	42	↑ TC: HR 0.84 (95% CI 0.78–0.92) ↑ LDL-C: HR 0.86 (95% CI 0.79–0.97) TG or HDL-C not found to be risk factors
Liu, 2018^50^	Prospective	Chronic heart failure	308	36	↑ TC: HR 0.99 (95% CI 0.97–1.00) ↑ LDL-C: HR 0.98 (95% CI 0.97–1.00) HDL-C not found to be risk factor
Ulus, 2018^51^	Prospective	Elderly (>65 years) with ACS undergoing PCI	308	NA	↑ MHR: OR 1.10 (95% CI 1.05–1.15)
Kim, 2018^52^	Retrospective	Community-based cohort of males	21 981	104	TG or HDL-C not found to be risk factors
Kokubo, 2017^53^	Prospective	Community-based cohort	6898	166	TC, TG or HDL-C not found to be risk factors
Aronis, 2017^54^	Prospective	Community-based cohort	9908	167	↑ Lp(a) not found to be risk factor
Saskin, 2017^55^	Retrospective	Isolated CABG	662	0.23	↑ MHR: OR 11.5 (95% CI 1.25–106.67)
Krittayaphong, 2016^56^	Retrospective	Hypertensive	13 207	NA	↑ LDL-C: OR 0.53 (95% CI 0.37–0.78)
Alonso, 2014^57^	Prospective	Community-based cohort	7142	115	↑ HDL-C: HR 0.64 (95% CI 0.48–0.87) ↑ TG: HR 1.60 (95% CI 1.25–2.05) TC and LDL-C not found to be risk factors
Mora, 2014^58^	Prospective	Healthy female healthcare professionals	23 738	197	↑ LDL-C: HR 0.72 (95% CI 0.56–0.92) ↑ VLDL-particles: HR 0.78 (95% CI 0.61–0.99) ↑ LDL-particles: HR 0.77 (95% CI 0.60–0.99) ↑ Cholesterol-poor small LDL: HR 0.78 (95% CI 0.61–1.00) ↑ Small VLDL particles: HR 0.78 (95% CI 0.62–0.99) Larger cholesterol-rich LDL-particles, total HDL-C, Lp(a) and TG not found to be risk factors
Lopez, 2012^59^	Prospective	Community-based cohort	13 044	224	↑ LDL-C: HR 0.90 (95% CI 0.85–0.96) ↑ TC: HR 0.89 (95% CI 0.84–0.95) HDL-C, TG and use of lipid-lowering medications not found to be risk factors
Watanabe, 2011^60^	Prospective	Community-based cohort	28 449	54	↑ HDL-C in females: HR 0.35 (95% CI 0.18–0.67) ↑ HDL-C in males not found to be risk factor: HR 0.74 (95% CI 0.42–1.30) ↑ TC: HR 0.94 (95% CI 0.90–0.97) ↑ LDL-C: HR 0.92 (95% CI 0.88–0.96)
Iguchi, 2010^61^	Prospective	Community-based cohort	30 449	NA	Hypercholesterolaemia, as defined by TC >220 mg/dL or the use of cholesterol-lowering agents: OR 0.75 (95% CI 0.58–0.96)
Haywood, 2009^62^	Prospective	Hypertensive	39 056	NA	↑ HDL-C: OR 0.77 (95% CI 0.62–0.95)
Rosengren, 2009^63^	Prospective	Community-based cohort of males	6903	412	TC not found to be risk factor
Frost, 2005^64^	Prospective	Population-based cohort without endocrine or cardiovascular diseases at baseline	47 589	68	(Females) ↑ TC: HR 0.57 (95% CI 0.42–0.78) TC not found to be a risk factor in males

ACS, acute coronary syndrome; AF, atrial fibrillation; CABG, coronary artery bypass graft; CI, confidence interval; HDL-C, high-density lipoprotein cholesterol; HR, hazard ratio; LDL-C, low-density lipoprotein cholesterol; Lp(a), lipoprotein(a); MHR, monocyte- to high-density lipoprotein cholesterol ratio; NA, not applicable; OR, odds ratio; PCI, percutaneous coronary intervention; PR, prevalence ratio; STEMI, ST-elevation myocardial infarction; TC, total cholesterol; TG, triglycerides; VLDL-C, very-low-density lipoprotein cholesterol.

A health survey performed by Iguchi *et al.*^61^ found that hypercholesterolaemia, defined by TC >220 mg/dL or the use of cholesterol-lowering agents, was related to reduced new-onset AF. Reduced levels of LDL-C has also been linked to increased prevalence of AF.^56^ In one study of 88 785 patients, for example, TC and LDL-C levels were inversely linked to incident AF over a follow-up period of 7 years.^48^ The authors reported no significant association between incident AF, and HDL-C or TG. However, the overall incidence of AF was extremely low at 0.52 per 1000 person-years.^48^ Similar findings were described in the ARIC (Atherosclerosis Risk in Communities) cohort which was validated even when analysing lipid levels as time-dependent variables.^59^ An ancillary study to ALLHAT (Antihypertensive and Lipid-Lowering Treatment to Prevent Heart Attack Trial) demonstrated that low HDL-C was associated with a significant increase in incident AF.^62^ In a Japanese cohort, Watanabe *et al.*^60^ also found that both TC and LDL-C were inversely related to incident AF. Furthermore, reduced levels of HDL-C was independently associated with greater incidence of AF in females, but not males. The former had a 28% higher risk of AF with each 10% decrease in HDL-C. Results from the SPCCD (Swedish Primary Care Cardiovascular Database) showed that each unit (mmol/L) increase in TC and LDL-C were associated with a 19% and 16% lower risk of incident AF, respectively; also, HDL-C and TG were not related to incident AF. In contrast to the previous study, Mourtzinis *et al.*^49^ found no sex-specific differences in outcomes based on lipid abnormalities.

The relationship (or lack of) between the aforementioned measures of lipid abnormalities and incident AF has also been demonstrated among patients with ST-elevation myocardial infarction^46^ and chronic heart failure.^50^ In a small study of patients who had AF ablation, TC and LDL-C were inversely associated with a higher risk of AF recurrence.^65^ However, subgroup analysis demonstrated that these factors were only significant in females but not males. The levels of HDL-C and TG were not related to AF recurrence post-ablation.^65^ The inverse relationship between AF, and TC and LDL-C are further supported by the fact that use of lipid-lowering medications does not reduce the risk of incident AF.^59^^,^^66^

It is worth noting that conflicting results have been demonstrated in few studies. A combined analysis of the MESA (Multi-Ethnic Study of Atherosclerosis) and Framingham Heart Study cohorts found that raised HDL-C and TG were independently associated with a lower risk of new-onset AF.^57^ However, the authors reported that TC and LDL-C were not important risk factors for new-onset AF. In a community-based cohort of Korean males, Kim *et al.*^52^ found that although the presence of metabolic syndrome led to greater incidence of AF over a follow-up period of 8.7 years, this was driven primarily by central obesity, and neither TG or HDL-C were risk factors for incident AF. Similar results were obtained from a historical Japanese population.^53^

Different study designs, populations, lifestyles and age ranges may partly explain some of the inconsistencies of previous studies. Nonetheless, the current literature strongly indicates that both TC and LDL-C have an inverse relationship with incident AF. This is supported by a recent meta-analysis of nine large cohort studies.^67^ Overall, these findings are important as they imply that a reduction in TC and LDL-C, may have unintended consequences for the risk of incident AF. The role of TG and HDL-C, and whether there are sex-specific responses to lipid abnormalities with regards to AF need further investigation.

In addition to the measures of lipids described above, several others have been explored in relation to incident AF. Aronis *et al.*^54^ found that Lp(a) levels above 50 mg/dL (compared to <10 mg/dL) were not associated with incident AF. Monocyte to HDL-C ratio has also been described as a novel biomarker of inflammation that may be useful to predict new-onset AF in patients undergoing percutaneous coronary intervention^51^ or coronary artery bypass grafting.^55^

### 3.2 Underlying mechanisms

In general, there is limited research on mechanisms that underpin the relationship between lipoproteins and AF. In a report from the Women’s Health Study, Mora *et al.*^58^ conjectures that the inverse relationship may be due to the stabilizing effect of cholesterol on myocardial cell membranes. This may occur through the effects of cholesterol on the regulation of ion channels and sensitivity of volume-regulated anion current to osmotic gradients.^68–71^ Furthermore, cholesterol depletion has been found to impair cardiomyocyte contractility by deregulation of calcium handling, adrenergic signalling and the myofibrillar architecture.^72^

The link between cholesterol levels and development of AF may also be related to inflammation. It has been shown that TC, LDL-C and HDL-C levels were decreased while TG was increased during inflammation.^73^ Therefore, reduced levels of cholesterol may be reflective of underlying inflammatory processes within the host that contributes to AF. Furthermore, lipoproteins influence the course of sepsis by binding to bacterial endotoxins and attenuate the harmful effects of inflammatory responses.^74^

It was reported that the effects of lipoproteins on incident AF extended beyond the cholesterol content to include the number of lipoprotein particles for LDL and VLDL.^58^ In this regard, it was the smaller particles for each of these lipoproteins that were the actual driving force contributing to the inverse relationship with AF as larger cholesterol-rich LDL-particles, total HDL-C, Lp(a) and TG were not associated with incident AF.^58^ In a small study of female patients undergoing catheter ablation, those with AF had smaller lipoprotein particles with increased oxidation, glycation and TG content compared to controls in sinus rhythm.^75^ Similar findings have been reported elsewhere among male patients.^76^ Overall, these changes resulted in enhanced foam cell formation via accelerated phagocytosis by macrophages, and reduced antioxidant ability of HDL.^75^ These changes are important as HDL particles have been shown to be more protective against cardiovascular events,^77^^,^^78^ which are known to contribute to AF. Furthermore, foam cells are known to initiate a wide range of bioactivities including inflammatory processes^79–81^ that may be linked to the pathogenesis of AF.

Sex differences in the association of lipoproteins and AF that were observed in some studies may be attributable to hormones, especially oestrogen, and differences in body fat distribution or insulin sensitivity.^82–84^ Moreover, a fall in testosterone levels among ageing males may influence oxidative modification of LDL-C.^85^

**Figure 4:**
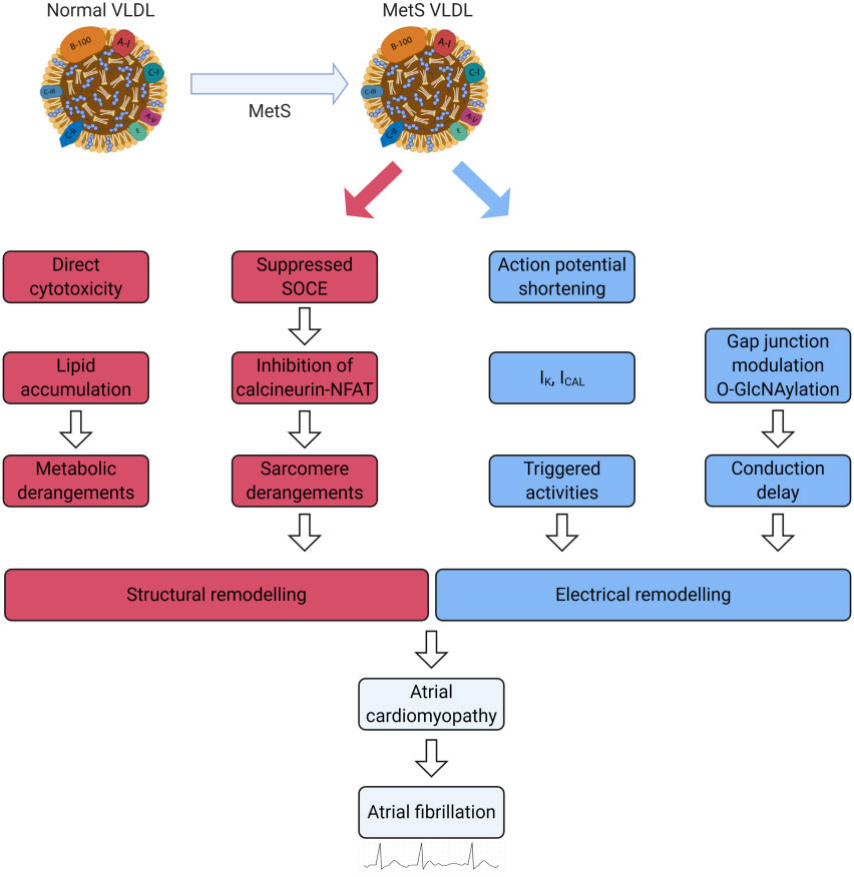
Pathogenic role of VLDL in metabolic syndrome-related atrial cardiomyopathy. Created using Biorender.com. MetS, metabolic syndrome; NFAT, nuclear factor of activated T cells; SOCE, store-operated calcium entry; VLDL, very low-density lipoprotein.

It is worth mentioning that the effects of specific lipoproteins may vary under certain conditions. For example, injection of VLDL extracted from patients with metabolic syndrome into mice resulted in excess lipid accumulation and apoptosis in the atria, and significantly greater left atrial dilatation compared to VLDL from healthy volunteers.^86^ Thus, VLDL may contribute to the development of atrial cardiomyopathy and subsequent vulnerability to AF through direct cytotoxicity, altered action potentials, disrupted calcium regulation, delayed conduction velocities, modulated gap junctions and derangements in sarcomere proteins (*Figure 4*).^87^ This highlights the fact that focusing on the quantity of lipoproteins on its own may limit our understanding of the mechanisms underlying the paradoxical inverse relationship of lipoproteins and AF.

## 4. Lipoproteins and thrombosis

The role of lipoproteins in modulating thrombosis and haemostasis to produce fibrin clots is well described.^88^ LDL and VLDL have been shown to increase thrombin generation and inhibit fibrinolysis.^89^^,^^90^ An inverse relationship of VLDL to fibrin clot permeability and fibre mass-length ratio has previously been demonstrated.^91^

In addition to the coagulation system, platelets seem to be affected by lipoproteins as well. To start with, there is evidence that patients with excessive LDL, such as those in familial hypercholesterolaemia that is characterized by lack or defective LDL receptors, display enhanced platelet reactivity with increased α-granule secretion,^92^ fibrinogen binding,^93^ and aggregation.^94^ In contrast, patients with abetalipoproteinaemia that is characterized by a lack of all apolipoprotein B-containing lipoproteins (chylomicrons, VLDL and LDL) have reduced platelet activation.^95^ Furthermore, LDL has been shown to promote excess platelet activation which may contribute to the higher incidence of thrombosis in hyperlipidaemia.^96^^,^^97^

Certain subclasses of LDL may be more harmful than others. For instance, sdLDL was shown to be independently associated with both thrombotic and haemorrhagic strokes.^98^ A potential mechanism could include increased susceptibility to oxidation which leads to a substantial increase in thrombin generation compared to the larger native LDL.^99^^,^^100^ In addition to identifying the lipid subclasses and oxidative states, evaluating the effects of individual lipid species may be of importance. For instance, Klein *et al.*^101^ demonstrated that VLDL was capable of activating the contact pathway in the presence of platelets, thereby causing an increase in the rate and amount of thrombin generation. A subsequent detailed lipoprotein analyses revealed that this was driven by phosphatidylethanolamine (PE). Interestingly, PE is also responsible for oxLDL-induced thrombin generation.^102^

### 4.1 OxLDL and haemostasis

Despite many decades of research into oxLDL, definitions of what it contains and method of detection vary between groups and publications.^33^ Perhaps the most encompassing definition for oxLDL is ‘A particle derived from circulating LDL that may have peroxides or their degradation products generated within the LDL molecule or elsewhere in the body associated with the particle’.^33^ Such particles therefore may include lipid peroxides, hydroxides or aldehydes such as malondialdehyde in addition to protein oxidation products. These biochemical changes give oxLDL altered properties which may facilitate its detection and separation on the basis of density, negative charge and monoclonal antibody (mAb). The latter method utilizes antibodies to oxidized epitopes on the surface of oxLDL such as EO6 for oxidized phosphatidylcholine (oxPC)^103^ and 4E6 for oxidized apoB.^104^ Given the variation in detection methods of oxLDL and possible consequences on interpretation of the evidence, this review specifies the method of detection of oxLDL where appropriate.

Elevated oxLDL levels (detected by 4E6 mAb) are independently associated with several cardiovascular risk factors including increasing age, male gender, raised body mass index, abdominal obesity, hypertension, raised C-reactive protein, renal dysfunction, hyperuricaemia, and smoking.^105^ These risk factors are important in AF, which has also been shown to be directly associated with elevated 4E6-measured oxLDL levels.^106–109^

Oxidized LDL (4E6 mAb) correlates to thrombogenesis by interfering with the coagulation system and clot formation. In this regard, patients with acute coronary syndrome demonstrate a positive correlation between oxLDL and tissue factor levels in plasma.^110^ Activation of T lymphocytes by oxLDL, prepared by chemical oxidation of native LDL with copper sulfate, via the lectin-type oxLDL receptor 1 (LOX-1) has also been shown to increase the expression of tissue factor on the surface of leukocytes.^111^ Furthermore, oxLDL generated with copper oxidation was noted to inhibit fibrinolysis, modify fibrin clot structure and increase thrombin generation.^102^^,^^112^ Finally, oxLDL (detected by 4E6) correlated to reduced clot permeability and prolonged clot lysis time.^113^

OxLDL generated *in vitro* by copper oxidation has been shown to cause activation and aggregation of platelets via CD36 and LOX-1,^114–116^ as well as impair endothelial regeneration by reducing the release of nitric oxide.^117^ Furthermore, platelet reactivity in cardiovascular disease can be related to dyslipidaemia,^118^^,^^119^ which is characterized by accumulation of oxLDL as measured by LDL isolation, lipid extraction and subsequent high performance liquid chromatography.^120^ In turn, platelet reactivity is an important determinant of fibrin clot structure and effective platelet inhibition is associated with a weaker, more permeable fibrin network.^121^ Therefore, oxLDL may indirectly influence fibrin clot properties through its effects on platelet reactivity. To complicate matters, recent evidence suggests that oxLDL activation of platelets promotes further oxLDL uptake by platelets (detected with the polyclonal orb10973 anti-oxLDL antibody), augmenting the pro-oxidative thrombogenic phenotype.^122^ Finally, there is evidence suggesting that activated platelets contribute to the formation of oxLDL species and modification of lipoprotein function.^123^ Putting it together, the evidence points towards a cycle of oxLDL-induced platelet activation leading to further oxLDL formation and uptake by platelets.

### 4.2 Lp(a) and haemostasis

In addition to its recognized atherogenic properties,^124^ Lp(a) appears to have a direct prothrombotic effect by interfering with platelets and the fibrinolysis system. Although it has been found to interact with platelets, the target receptor remains unclear.^125^ Furthermore, literature surrounding the nature of interaction between Lp(a) and platelets is conflicting, with evidence to suggest that it may have both activating and inhibiting effects.^126^

Lp(a) has been shown to facilitate platelet activation through thrombin-related activating hexapeptide, but not thrombin or adenosine diphosphate.^127^ On the contrary, some studies reported an inhibitory effect of Lp(a) to platelet activation by collagen or thrombin.^125^ Less controversial is the ability of Lp(a) to impair platelet-mediated fibrinolytic reactions by interfering with the binding of plasminogen, which shares structural similarities to apolipoprotein(a), and tissue plasminogen activator to the platelet surface.^128^ This is compounded by the ability of Lp(a) to inactivate tissue factor pathway inhibitor which may promote thrombosis through the extrinsic coagulation pathway.^129^ However, evidence in genetic studies on the contribution of Lp(a) to venous thrombosis have been negative,^130^^,^^131^ suggesting that the primary prothrombotic effects of Lp(a) may be limited to atherothrombosis (arterial) or anti-fibrinolysis.^132^ Additional studies describing the association between lipoproteins and thrombotic conditions are summarized in *Table 2*.

**Table 2:** Clinical studies describing association of lipoproteins with thrombotic conditions

Author, year (ref)	Study design	Population	*n*	Finding(s) in relation to thrombosis
Morelli, 2017^133^	Case-control	Recent venous thrombosis	5107	↓ ApoB: OR 1.35 (95% CI 1.12–1.62) ↓ ApoA1: OR 1.50 (95% CI 1.25–1.79)
Grifoni, 2012^134^	Cross-sectional	First episode venous thromboembolism	747	↑ Lp(a): OR 2.6 (95% CI 1.7–4.0)
Kamstrup, 2012^131^	Community-based cohort	White Danish descent	41 231	↑ Lp(a): OR 1.21 (95% CI 1.10–1.33) for risk of myocardial infarction (coronary atherothrombosis) No association between Lp(a) and venous thrombosis
Ohira, 2006^135^	Cohort	No history of stroke	14 448	↑ Lp(a): OR 1.42 (95% CI 1.10–1.83) for non-lacunar strokes, No association between Lp(a) and lacunar or cardioembolic strokes
Tsimikas, 2005^35^	Cross-sectional	Coronary artery disease	504	↑ oxLDL: ApoB100 ratio: OR 3.12 (*P* < 0.01) ↑ Lp(a): OR 3.64 (*P* < 0.01)
Deguchi, 2005^136^	Cross-sectional	Men with venous thrombosis	98	↓ HDL: OR 6.5 (2.3–19) ↓ ApoA1: OR 6.0 (2.1–17) ↑ IDL: OR 2.7 (1.0–6.8, *P* < 0.05) ↑ sdLDL: OR 3.1 (1.3–7.4)
Doggen, 2004^137^	Case-control	Post-menopausal women with first venous thrombosis	2463	↑ HDL-C: OR 0.71 (95% CI 0.52–0.97) ↑ TG: OR 2.13 (95% CI 1.34–3.37)
Marcucci, 2003^138^	Case-control	History of venous thromboembolism	1033	↑ Lp(a): OR 2.1 (95% CI 1.4–3.2)
von Depka, 2000^139^	Case-control	History of venous thrombo-embolism	951	↑ Lp(a): OR 3.2 (95% CI 1.9–5.3)
Holvoet, 1998^140^	Case-control	Coronary artery disease	270	↑ oxLDL in acute coronary syndrome than stable angina (*r*^2^ 0.65, *P* < 0.01)
Kawasaki, 1997^141^	Case-control	Confirmed deep vein thrombosis	218	↑ TC: OR 4.5 (95% CI 2.4–8.3) ↑ TG: OR 2.4 (95% CI 1.3–4.6)

ApoA1, apolipoprotein A1; ApoB, apolipoprotein B; CI, confidence interval; HDL, high-density lipoprotein; HDL-C, high-density lipoprotein cholesterol; IDL, intermediate-density lipoprotein; Lp(a), lipoprotein(a); OR, odds ratio; OxLDL, oxidized low-density lipoprotein; sdLDL, small dense low-density lipoprotein; TC, total cholesterol; TG, triglycerides.

### 4.3 The effects of lipid-modifying therapy on thrombosis and haemostasis

The role of lipoproteins in haemostasis is further supported by the fact that application of lipid-modifying therapy is associated with changes in haemostasis.^142^ Specifically, atorvastatin may exert antiplatelet effects by interfering with redox signalling.^143^ It has also been shown that statins are able to reduce fibrin clot lysis time, independent of warfarin.^144^ For example, a randomized controlled trial by Undas *et al.*^145^ confirmed the effects of statins and also showed similar results with the use of other lipid-modifying therapy, specifically fenofibrate. The authors reported increased fibrin clot permeability and reduced lysis time with the use of these agents compared to pre-treatment values, potentially through its effects on thrombin generation. Turbidity analysis also showed that use of these drugs resulted in thicker fibres that were more prone to effective fibrinolysis.

A further randomized controlled trial of patients with type 1 diabetes mellitus and dyslipidaemia found that the beneficial effects of statins on fibrin clot properties may be related to reduced expression of glycoprotein IIIa, tissue factor, and P-selectin.^146^ Finally, the use of statins has been associated with risk reduction of both venous and arterial thromboembolisms.^147–151^ Therefore, it is tempting to speculate that the statin-induced protective effects may be related to its influence on reduction of pro-coagulant lipoproteins or enhancement of anti-coagulant lipoproteins.^90^

A prospective, case-controlled study of patients with stable coronary artery disease and hypercholesterolaemia found that use of pravastatin was associated with reduced thrombus formation at both high and low shear rates.^152^ As expected, there was a significant decrease in TC and LDL-C levels with pravastatin. Thrombus formation was also assessed after 1 week of treatment with pravastatin, prior to any significant reduction in TC and LDL-C levels, and it was found that this was unchanged compared to pre-treatment. As a result, the authors concluded that the beneficial effects of pravastatin on thrombogenicity was due to its effects on lipids/lipoproteins.^152^ Interestingly, other studies have reported that the anti-coagulant effects of statin therapy, in terms of thrombin generation and platelet activation, were seen as early as 3 days following treatment.^153^^,^^154^

Nonetheless, it should be noted that there currently remains insufficient evidence to conclude whether the protective effects of statins are related to its lipid-modifying effects or otherwise.^148^ In contrast to the aforementioned studies, Dangas *et al.*^155^ showed a reduction in thrombogenicity among patients after 6 months of treatment with pravastatin, regardless of change in LDL-C. Furthermore, despite a similar reduction in LDL-C between subgroups of patients treated with pravastatin compared to dietary advice only, the anti-thrombotic benefit was only demonstrated among those receiving pravastatin. Additionally, a study by Undas *et al.*^156^ found that the use of simvastatin was associated with a reduction in thrombin generation, independent of changes in lipid profile. Overall, there may be various pathways by which lipid-modifying therapy, in particular statins, may interact with the haemostatic process.

## 5. Lipoproteins and thromboembolism in AF

Given the effects of lipoproteins on haemostasis, their contribution to thromboembolic events may be expected. Indeed, lipoprotein abnormalities have been shown to be an independent risk factor for stroke and venous thromboembolism.^157–160^ However, few studies have explored this relationship in the context of AF (*Table 3*).

**Table 3:** Effects of lipoproteins on thromboembolic outcomes in atrial fibrillation

Author, year (ref)	Study type	Population	*n*	Follow-up (months)	Finding(s)
Liu, 2020^161^	Retrospective	Non-valvular AF	2345	26	↑ LDL-C in low-risk: HR 2.60 (95% CI 1.26–5.37) for ischaemic stroke ↑ LDL-C in high-risk: HR 2.50 (95% CI 1.10–5.70) for ischaemic stroke
Yan, 2019^162^	Retrospective	Non-valvular AF with low CHA_2_DS_2_-VASc score	595	NA	↑ Lipoprotein(a): OR 1.02 (95% CI 1.01–1.03) for thromboembolic events
Pol, 2018^163^	Prospective	AF with at least 1 stroke/SE risk factor	14 884	23	↑ Apolipoprotein A1: HR 0.81 (95% CI 0.73–0.90) for composite risk of ischaemic stroke, SE, MI and CV death Apolipoprotein B was not associated with composite risk of ischaemic stroke, SE, MI and CV death
Qi, 2017^164^	Retrospective	AF ± ischaemic stroke	815	NA	↑ LDL-C: OR 2.00 (95% CI 1.62–2.47) for ischaemic stroke
Aronis, 2017^54^	Prospective	Community-based cohort	10 127	190	↑ Lipoprotein(a) was not associated with stroke risk in patients with AF
Wu, 2017^165^	Retrospective	Non-valvular AF	2470	NA	↑ LDL-C: OR 1.27 (95% CI 1.08–1.49) for ischaemic stroke
Igarashi, 1998^166^	Prospective	Chronic AF	150	NA	↑ Lipoprotein(a) was an independent risk factor for LA thrombus (standardized coefficient of 0.300)

AF, atrial fibrillation; CI, confidence interval; CV, cardiovascular; HR, hazard ratio; LA, left atrial; LDL-C, low-density lipoprotein cholesterol; MI, myocardial infarction; NA, not applicable or available; OR, odds ratio; SE, systemic embolism.

### 5.1 Low-density lipoprotein cholesterol

LDL-C has been implicated in thromboembolic events among patients with AF. Wu *et al.*^165^ found that LDL-C was an independent risk factor for both a history of ischaemic stroke and future stroke risk among patients with AF. Similar findings were reported in a case-controlled study, whereby raised LDL-C was shown to be an independent predictor of ischaemic stroke in patients with AF, irrespective of the CHA_2_DS_2_-VASc score.^164^ Furthermore, this association demonstrated a dose-response pattern. A later study confirmed the relationship between LDL-C and ischaemic stroke, and observed that lowering LDL-C may be particularly beneficial among AF patients with a low CHA_2_DS_2_-VASc score (less than two in males and three in females).^161^ Interestingly, LDL-C appears to have an opposite influence on the risk of incident AF and subsequent thromboembolic risk which highlights the importance of regular monitoring and treatment adjustments in clinical practice.

### 5.2 Lipoprotein(a)

There are conflicting reports on the effects of Lp(a) on thromboembolic risk in AF. Igarashi *et al.*^166^ demonstrated that serum Lp(a) was an independent risk factor for left atrial thrombus detected on trans-oesophageal echocardiogram in patients with chronic AF. Additionally, left atrial thrombus was present in 48% of AF patients with a Lp(a) level ≥30 mg/dL, suggesting that this may be a useful biomarker to identify patients at high risk of thromboembolism. However, a limitation of this study was that relatively few patients (19%) were on anticoagulation therapy.^166^

More recently, higher Lp(a) levels were found to be independently associated with clinically confirmed thromboembolic events in non-valvular AF patients with a CHA_2_DS_2_-VASc score of less than two.^162^ Curiously, Aronis *et al.*^54^ found that elevated levels of Lp(a) was associated with an increased stroke risk among non-AF patients, but not in those with AF. In support of the latter, we previously demonstrated that there was no correlation between Lp(a) and D-dimer, as a marker of thrombogenesis.^167^ Overall, the inconsistent results on Lp(a) may suggest the existence of different Lp(a) phenotypes that contribute differently to thrombogenesis^168^ and therefore, sole measurement of total Lp(a) levels may be inadequate for this purpose. In this regard, the measurement of oxidized lipids may have an important role to increase our understanding on the potential impact of Lp(a) on atrial function and risk of AF.^169–171^

### 5.3 Other measures of lipoproteins

In a sub-study of the ARISTOTLE trial, higher levels of Apolipoprotein A1 were independently associated with a lower composite risk of ischaemic stroke, systemic embolism, myocardial infarction, and cardiovascular mortality.^163^ When analysed separately, Apolipoprotein A1 was found to be a risk factor for each of the individual outcomes apart from myocardial infarction. In reverse, the authors reported that Apolipoprotein B was not associated with the risk of composite outcomes but that it was a risk factor for myocardial infarction. Decker *et al.*^172^ demonstrated that low HDL and high TG were not independently associated with ischaemic stroke among AF patients over a follow-up period of 14.8 years, though there was a trend for the former [hazard ratio (HR) 1.47, 95% confidence interval (CI) 0.99–2.20; *P *=* *0.06].

The relationship between lipoproteins and thromboembolism in AF is further indicated by studies that have explored the impact of statins, as medications that are known to regulate lipoproteins. A subgroup analysis comprising of 1446 AF patients with ischaemic stroke found that higher statin adherence during 5-year follow-up predicted a reduced risk of stroke recurrence [HR 0.59 (95% CI 0.43–0.81)].^173^ In this context, the effects of statins may be related to a reduction of oxLDL levels that promote its anti-inflammatory properties,^174^^,^^175^ which has been shown to reduce the endogenous thrombin potential in patients with AF.^176^ He *et al.*^177^ found that prior use of statins resulted in lower plasma oxLDL levels at baseline and at 3-month follow-up among patients presenting with an ischaemic stroke. Furthermore, pre-stroke statin use was associated with reduced short-term mortality [odds ratio (OR) 0.38 (95% CI 0.16–0.91) and major disability (OR 0.38 (95% CI 0.15–0.99)].

## 6. Gaps and limitations

Despite a wealth of evidence on the role of lipoproteins in thrombosis and AF, it is recognized that these molecules are heterogeneous, containing numerous subclasses and lipid species with variable effects.^178^ In this regard, much of the conflicting evidence and paradox in prior studies may be due to the usage of crude methods of classification that undermines the complexity of lipoproteins. Given recent advancements in our ability to accurately analyse lipoprotein subclasses and lipid species, future studies should focus on identifying the relationship of these molecules with incident AF and thromboembolic complications. Moreover, the mechanism by which this occurs also warrants further investigation. With better understanding in this area, the development of targeted treatment approaches for high-risk subgroups may be possible. Furthermore, ongoing clinical trials such as the Lp(a)HORIZON study (ClinicalTrials.gov NCT04023552) are examining novel agents targeting Lp(a) levels and may provide more data on the association of Lp(a), incident AF and thrombotic events.

One group of lipids which is emerging as a key player in haemostatic reactions is oxidized GPL. These molecules have been shown to play a role in thrombotic disorders and are primarily generated enzymatically by platelets and leukocytes.^179^^,^^180^ The presence of these molecules in lipoproteins has not been conclusively studied, particularly in light of newer lipidomic technologies. The majority of previous studies of oxidized GPL in lipoproteins had relied on antibodies that bind oxPC, demonstrating their presence as a defining feature of oxLDL^181^ and Lp(a).^182^ It is not known whether the presence of oxPC, or other oxidized GPL, on lipoproteins enhance coagulation reaction in a similar way to enzymatically-generated oxPC on the surface of activated cells.^179^ The growth in the lipidomics field and availability of increasingly sensitive techniques may pave the way for studies in this area.

Moving forward, the role of genetics in lipoproteins should also be considered. Elevated Lp(a) is prevalent in approximately 20% of the population,^183^ and strongly influenced by genetic variability.^184^ Much of the variation is related to the apo(a) protein, which consists of kringle domains that vary in molecular weight and therefore size of the Lp(a) particle.^185^^,^^186^ The genetic variation in the *LPA* locus has enabled Mendelian randomization studies to demonstrate that both the Lp(a) concentration and the smaller apo(a) isoform are independently causal for some cardiovascular diseases.^183^^,^^187–191^ While a large UK-based population study by Zanetti *et al.*^188^ found no causal relationship between Lp(a) and AF, further Mendelian randomization studies are needed to confirm this finding in other cohorts.

## 7. Conclusion

There is a paradoxical relationship between TC and LDL-C, and incident AF. The mechanism by which this occurs is poorly defined but may be related to changes in the regulation of ion channels and inflammatory processes. To complicate matters, excess lipoproteins promote thrombin generation, inhibit fibrinolysis and enhance platelet activation. In this regard, LDL-C has been shown to be an independent risk factor for thromboembolic events in AF. Overall, the complex relationship between lipoproteins, thrombosis and AF warrants further research. An improved knowledge base in this area may unlock important mechanistic pathways that contribute to our overall understanding of haemostasis and guide our clinical approach in the treatment of prothrombotic conditions.

## Data availability

The data that support the findings of this review are available from the corresponding author upon reasonable request.
